# Verification of the Functional Antioxidant Activity and Antimelanogenic Properties of Extracts of* Poria cocos* Mycelium Fermented with Freeze-Dried Plum Powder

**DOI:** 10.1155/2019/9283207

**Published:** 2019-06-02

**Authors:** Kyung-Yun Kang, Yun-Ho Hwang, Sung-Ju Lee, Ho-Yeol Jang, Seong-Gyeol Hong, Seul-Ki Mun, Su-Jin Kim, Jong-Jin Kim, Kuyng-Wuk Park, Kyoung-Sun Seo, Seung-Eon Ban, Seong-Woo Jin, Hyuck-Joo Kim, Sung-Tae Yee

**Affiliations:** ^1^Suncheon Research Center for Natural Medicines, Suncheon, Republic of Korea; ^2^Department of Pharmacy, Sunchon National University, Suncheon 540-742, Republic of Korea; ^3^Center for Self-Assembly and Complexity, Institute for Basic Science (IBS), Pohang 37673, Republic of Korea; ^4^Jangheung Research Institute for Mushroom Industry 288, Woodlannd-gil, Gisan-ri, Anyang-myeon, Jangheung-gun, Jeollanam-do 529-805, Republic of Korea; ^5^Department of Industrial Machinery Engineering, Sunchon National University, Suncheon 540-742, Republic of Korea

## Abstract

Here we examine the effects of extracts of* Poria cocos* mycelium fermented with freeze-dried plum powder (PPE) on the *α*-melanocyte stimulating hormone (*α*-MSH)-stimulated melanogenesis in cultured murine B16 melanoma cells (B16 cells), relative to the effects of Prunus extract. We found that an extract of Prunus fermentation showed significant inhibition of melanogenesis and tyrosinase activity with no effect on cell proliferation and was more active compared to Prunus extract alone. Furthermore, we confirmed that medium containing 3% Prunus was the optimal culture substrate for fermentation with* Poria cocos*. These results provide evidence that Prunus fermentation extract affects skin whiting in murine B16 melanoma cells (B16 cells). Prunus contains rutin, oxalic acid, succinic acid, and fumaric acid, which help in digestion and fatigue recovery. The rutin of* Prunus mume* is reported to have antioxidant and anti-inflammatory effects. Also, Prunus extract has a tyrosinase inhibitory activity for skin whiting through its antioxidant activity. Therefore, we believe the Prunus extract for* Poria cocos* fermentation can be provided as a potential mediator to induce skin whiting.

## 1. Introduction

Melanin plays a key role in photoprotection and imparts skin color. It is well documented that overproduction and excessive accumulation of melanin leads to various human skin disorders, such as melasma, freckles, age spots, and malignant melanomas [1]. Melanin synthesis is modulated by the number of melanocytes present in the epidermis, and the size and amount of melanosomes generated by melanogenic enzymes [2–5]. Among the many enzymes involved, melanogenic enzymes present in melanocytes and melanoma cells, such as tyrosinase, tyrosinase-related protein 1 (TRP-1), and tyrosinase-related protein 2 (TRP-2), are the most important regulators of melanin biosynthesis [2–9]. Tyrosinase is a rate-limiting enzyme of melanogenesis and exerts its effect by catalyzing the hydroxylation of tyrosine to 3, 4-dihydroxyphenylalanine (DOPA), and the oxidation of DOPA to DOPA-quinone. The TRP-1 complexes include TRP-1 (involved in the oxidization of 5,6-dihydroxyindole-2-carboxylic acid (DHICA) to a carboxylated indole-quinone) and TRP-2 (which functions as a DOPA-chrome tautomerase and catalyzes the rearrangement of DOPA-chrome to DHICA) [2–9]. Therefore, melanogenic enzymes, such as tyrosinase and the TRP-1 complex, are important in tyrosinase activation and stability. This has increased the demand of tyrosinase inhibitors for use in skin whitening agents – from synthetic [1, 2] or natural resources [1] – for both beauty and therapeutic purposes, which are currently under development [2–9]. Tyrosinase inhibitors have been widely studied for their reduction of melanogenesis [1–10].* Prunus mume* is known to have various biological activities and is widely cultivated in China, Japan, and Korea.* P. mume* is reported to contain oxalic acid, succinic acid, and fumaric acid, which help in digestion and fatigue recovery [11, 12]. The rutin of* P. mume* is reported to have antioxidant and anti-inflammatory effects. Also, the extract of Prunus was found to possess tyrosinase inhibitory activity for skin whiting by exerting its antioxidant effect [11, 12]. Also, the material for fermentation* Poria cocos* (Schw.) Wolf is a cluster of sclerotial bodies that include fungi and parasitic fungi (parasitic mushrooms) residual in the roots present under the ground 4–5 years after pine trees have been harvested [11, 12]. Depending on their color within and their origin, they are named as follows: white (Baekbokryeong), pink (Juckbokyeong), originating from pine roots (penetrated) [11, 12]. This study was undertaken to examine the tyrosinase inhibitory activity of the extract of Prunus fermentation. To address this inhibitory activity, the effects of Prunus fermentation extract were assessed for *α*-MSH-stimulated melanogenesis in B16 melanoma cells, relative to Prunus extract alone. This study further confirmed the optimum content of Prumus extract for* Poria cocos* (Schw.) Wolf mycelium fermentation.

## 2. Material and Methods

### 2.1. Chemicals

The following chemicals were procured from Sigma-Aldrich, St. Louis, MO, USA: ascorbic acid, 1,1-diphenyl,2-picryl hydrazyl (DPPH), gallic acid, vanillin, (+)-catechin, sulfuric acid, sodium dodecyl sulphate (SDS), sulfuric acid (H_2_SO_4_), 2,2′-azino-bis(3-ethylbenzothiazoline-6-sulfonic acid) diammonium salt, potassium ferricyanide (K_3_[Fe(CN)_6_]), ferrous sulphate (FeSO_4_), ferric chloride (FeCl_3_), sodium carbonate (Na_2_CO_3_), sodium nitrite (NaNO_2_), sodium hydroxide (NaOH), aluminum chloride (AlCl_3_), copper(II) chloride (CuCl_2_), iron(II) chloride (FeCl_2_), ethanolic neocuproine, Folin–Ciocalteu's phenol reagent, 2,2′-bipyridyl, ethylenediaminetetraacetic acid (EDTA), ammonium acetate, dimethyl sulfoxide (DMSO), and propidium iodide (PI). Potassium persulfate (Junsei, Japan), HPLC grade methanol, and ethanol (J.T Baker, U.S.A) were the other chemicals used.

RPMI 1640 medium was purchased from Thermo SCIENTIFIC, DMEM from Gendepot, and the cell counting Kit-8 (CCK-8) from Dojindo Laboratories.

### 2.2. Mycelium Culture and Fermentation of Material Extracts

Freeze-dried plum pulp was purchased from Suncheon N Plum Ltd. (Suncheon City, Republic of Korea) and extracted. Control is cultured media, PC1% (freeze-dried plum powder 1%+cultured media) and PP (*Poria cocos* mycelium fermented of culture media). The* Poria cocos* mycelia were cocultured with 0.1%, 0.3%, 1%, 3%, and 10% concentrations of the lyophilized powder of dried plums, and incubated for 9 days in a shaking incubator. The resultant mushroom mycelium culture was homogenized and mixed with 70% fermented alcohol at a ratio of 1:1 (V/V), followed by extraction for 1 day at 14°C in a shaking incubator. The extracts were filtered through Whatman filter and used as materials for all experiments. Samples were diluted to the required concentrations for further experiments.

### 2.3. Antioxidant Activity

#### 2.3.1. Chemical Composition: Phenolics, Tannins, and Flavonoids

Catechins and proanthocyanidins reactive to vanillin were analyzed using the vanillin method of Richard and William (1978) [13], with slight modification. Using a calibration curve, concentrations were calculated as g catechin equivalents (CE)/kg dry mass; the tannin concentration was expressed as mg CE/g.

The total flavonoid content was evaluated using the method of Thomas et al. (2012) [14], with slight modifications. Quercetin as a standard was evaluated at varying concentrations from 1-500 *μ*g/mL, to generate a calibration curve. The total flavonoid concentration was expressed as mg QE/g.

The total phenolic content method of Thomas et al. (2012) [14] and Zhang et al. (2006) [15] was used with slight modifications [16]. Gallic acid was used as a standard (1-500 *μ*g/mL) to produce a calibration curve. The total phenolic concentration was expressed as mg GAE/g.

#### 2.3.2. Radical and Anion Scavenging Activity

Antioxidant activity was studied using the 1,1-diphenyl-2-picrylhydrazyl free radical (DPPH) method as described by Blois (1958) [17] and Thomas et al. (2012) [14], with slight modifications. The ABTS cation radical scavenging activity of extracts was performed using the spectroscopic method described by Roberta et al. (1999) [18]. The superoxide radical scavenging activity of extracts was assessed by the protocol suggested by Zhishen et al. (1999) [19], with slight modifications [16], using ascorbic acid as the standard. Data are expressed as the mean values ± standard deviation (SD) of three measurements. All scavenging activities of each solution are calculated as percent inhibition, according to the following equation:

(1) $$\begin{array}{c} \text{Scavenging rate }\left(\;\%\;\right)=\frac{\left[A_{\left(blank\right)}\text{-}A_{\left(sample\right)}\right]}{A_{\left(blank\right)}}\times 100 \end{array}$$

#### 2.3.3. Fenton Reaction and Reducing Power Activity

The metal chelating ability of extracts were predicted according to the method of Dinis et al. (1994) [20], with slight modifications (Gülçin et al. 2007) [16, 21]. The Fe^2+^ chelating ability of each solution was calculated as a percent inhibition according to the following equation:

(2) $$\begin{array}{c} \text{Scavenging rate }\left(\;\%\;\right)=\frac{\left[A_{\left(blank\right)}\text{-}A_{\left(sample\right)}\right]}{A_{\left(blank\right)}}\times 100 \end{array}$$

The reducing power of Cu^2+^ was studied using the reducing ability method described by Apak et al. (2006) [22] and Gülçin (2008) [23], with slight modifications. Absorbance of samples was recorded at 450 nm after 30 min incubation (Gülçin. 2008) [23]. Extracts were also subjected to the FRAP assay following the method of Iris and Strain (1996) [24], with slight modifications. Absorbance of the mixture was measured at 593 nm (Göcer and Gülçin. 2011) [25]. The Fe^3+^ reducing assay measured the Fe^3+^ reducing ability of the extracts, using the Fe^3+^(CN^−^)_6_ to Fe^2+^(CN^−^)_6_ reduction method described by Gülçin (2007) [26] and Gülçin (2010) [27], with slight modifications [16]. Absorbance was measured at 700 nm using a spectrophotometer. It is well documented that increase in reduction capabilities results in increased absorbance [28, 29]. The data are the mean values ± standard deviation (SD) of three measurements.

### 2.4. Antimelanogenic Properties

#### 2.4.1. Cells and Cell Culture

Melanoma B16F0 cells (CRL-6322) were obtained from ATCC (Manassas, VA, U.S.A.) and cultured in Dulbecco's Modified Eagle Medium (DMEM) supplemented with 10% fetal bovine serum (FBS), 100 U/mL of penicillin G, and 100 *μ*l/mL of streptomycin sulfate. The purities of all standard compounds tested were confirmed to be >95% by HPLC. Samples of the test compounds were dissolved in dimethyl sulfoxide (DMSO) and added to the media at a final concentration of 0.03% DMSO. Cultures were maintained at 37°C under 5% CO_2_ / 95% air, and the media were changed every two days.

#### 2.4.2. Cytotoxicity Assay

Cell viability was determined using the cell counting Kit-8 (CCK-8) assay. Melanoma B16F0 cells were suspended in Dulbecco's Modified Eagle Medium (DMEM) at a density of 1×10^5^ cells/mL; 100 *μ*L aliquot of the cell suspension was added per well of 96-well flat-bottomed microtiter plates, followed by addition of 100 *μ*L of the test samples (final concentrations of Prunus fermentation extracts were 100, 500, and 1000 *μ*g/ml), and incubated at 37°C. After 24h, 10 *μ*L of CCK-8 solution was added per well and the plates were further incubated for 3h. Absorbance was detected at 450nm with a microplate reader. The cell viability is expressed as a percentage of the control culture.

#### 2.4.3. Melanin Content Measurement

To determine the amount of melanin produced, 1×10^6^ B16F0 cells were plated per well and exposed to 1000 *μ*M *α*-MSH for 1h, after which the cells were treated with 1000 *μ*g/ml Prunus fermentation extract for 48 h. The cells were collected by trypsinization, washed twice with PBS, air dried, and finally dissolved in 200 *μ*L of 1 M NaOH and maintained at 90°C for 30 min to dissolve the cell aggregates. The suspension was then centrifuged, and the resultant supernatant was assessed for melanin content. The quantity of melanin was determined as the absorbance at 450 nm using a spectrophotometer. Absorbance was compared to a standard curve of known synthetic melanin concentrations.

#### 2.4.4. Tyrosinase Activity

Tyrosinase activity was estimated by measuring the rate of oxidation of 3, 4-dihydroxy-L-phenyl-alanine (L-DOPA). About 1×10^6^ cells/well of the B160 cells were exposed to 1000 *μ*M *α*-MSH for 1h, following which the cells were treated with 1000 *μ*g/ml Prunus fermentation extract for 48 h. The cells were washed and then lysed in 300 *μ*l sodium phosphate buffer (0.1 M, pH 6.8) containing 0.1% (w/v) Triton X-100. The extract was clarified by centrifugation at 15000 g for 10 min at 4°C to obtain a crude enzyme tyrosinase solution in the supernatant; wells were seeded at 100 *μ*l/well concentration and treated with 100 *μ*l/well L-DOPA solution. After incubation at 37°C for 2 h, absorbance was measured at 490 nm using a spectrophotometer.

### 2.5. Statistical Analysis

Differences in the data between groups are presented as the mean ± S.D. of three replicates. Statistical differences were analyzed using the Student's t-test. Probability values less than 0.05 are considered to be significant (P values *∗* < 0.05, *∗∗* < 0.01, *∗∗∗* < 0.001).

## 3. Results and Discussion

### 3.1. Chemical Composition: Phenolics, Tannins, and Flavonoids

The total phenolic compounds, flavonoid content, and condensed tannin content of extracts of the cultured* Poria cocos* mycelium fermented with freeze-dried plum powder (PPE) were determined using gallic acid, quercetin, and catechin calibration curves, respectively.  Table 1 shows the concentrations of flavonoids, phenolic, and tannic compounds of the fermented extracts.

The total phenolic content in 3% PPE was 4.701 ± 0.006 *μ*g GAE/mg, flavonoids totaled 5.333 ± 0.001 *μ*g QE/mg, and condensed tannin totaled 21.000 ± 0.003 *μ*g CE/mg.

Higher levels of phenolics and flavonoids were confirmed in 3% PPE extracts as compared to other concentrations of PPE. Additionally, the active ingredients that aid antioxidation through fermentation were also confirmed to be higher in 3% PPE. Condensed tannin was detected only after fermentation and, thus, was thought to be formed through the metabolism or the fermenting microbes. These results indicate that flavonoids of natural products increase the antioxidant activities, such as the ability to donate electrons, in proportion to the content of phenolic materials (Table 1).

### 3.2. Determination of Antioxidant Activities

#### 3.2.1. Radical and Anion Scavenging Activity

**Figure 1:**
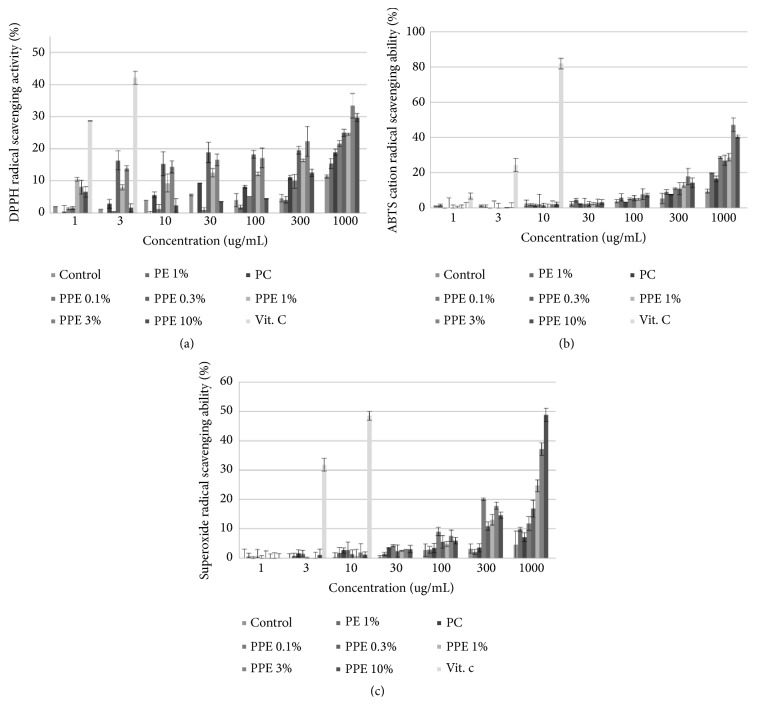
(a) The DPPH radical scavenging activity of extracts of* Poria cocos* mycelium fermented with freeze-dried plum powder (PPE). (b) The ABTS cation radical scavenging activity of extracts of* Poria cocos* mycelium fermented with freeze-dried plum powder (PPE). (c) The superoxide radical scavenging activity of extracts of* Poria cocos* mycelium fermented with freeze-dried plum powder (PPE). [Vit.C: ascorbic acid] Results are mean ± S.D. of triplicate data.

Figure 1 shows the concentrations of the DPPH radical, ABTS cation radical, and superoxide anion radical scavenging activity, respectively, found in the extracts of* Poria cocos* mycelium fermented with freeze-dried plum powder (PPE).

By the electron-donating ability assay, we found that 3% PPE has a 33.38% activity at 1000 *μ*g/mL. These results are consistent with studies that show increased DPPH radical scavenging activities when total polyphenol content is higher, relative to the total phenolic content and antioxidant activities in 3% PPE that have high total polyphenol and flavonoid contents. Also, the ABTS cation radical scavenging activities were found to be 47.14% at the 3% PPE concentration. This pattern is similar to that of the electron-donating abilities at a concentration of 1000 *μ*g/mL. The effects of the ABTS cation radical scavenging abilities and electron-donating abilities are presented in Figure 1. Conversely, at the same concentration, the NBT assay revealed superoxide anion radical scavenging activities to be 48.78% in 10% PPE. This result differs from the patterns of DPPH radical scavenging activities and ABTS cation radical scavenging activities; however, the values are not significantly different relative to those obtained for 3% PPE.

#### 3.2.2. Fenton Reaction and Reducing Power Activity

**Figure 2:**
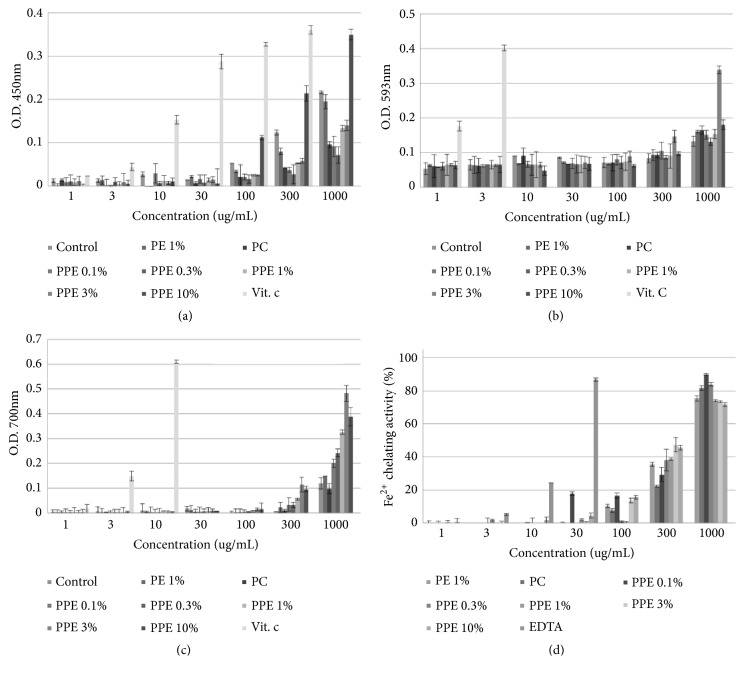
(a) The Cu^2+^ reducing ability of extracts of* Poria cocos* mycelium fermented with freeze-dried plum powder (PPE) and of reference antioxidants. [Vit.C: ascorbic acid] (b) The Fe^3+^ − TPTZ − Fe^2+^ − TPTZ reducing ability of extracts of* Poria cocos* mycelium fermented with freeze-dried plum powder (PPE) and of reference antioxidants. (c) The Fe^3+^→ Fe^2+^ reductive potential of different of extracts of* Poria cocos* mycelium fermented with freeze-dried plum powder (PPE) and of reference antioxidants. (d) The Fe^2+^ chelating of different of extracts of* Poria cocos* mycelium fermented with freeze-dried plum powder (PPE) and of reference antioxidants. Results are mean ± S.D. of triplicate data.

Figure 2 shows the concentrations of the Fenton reaction and reducing power activities found in the extracts of* Poria cocos* mycelium fermented with freeze-dried plum powder (PPE).

In the Fenton oxidation reaction, H_2_O_2_ and Fe^2+^ form an OH radical intermediate that bonds with organic compounds. To measure the reducing power in this oxidation reaction, we measured the reducing powers of Fe (Fe^2+^) and Cu (Cu^2+^), as well as the antioxidant activities of chelating reactions, which inhibit the formation of the Fe^2+^- ferrozine complex.

The FRAP assay is based on the principle that, at low pH, the ferric tripyridyl triazine (Fe^3+^- TPTZ) complex is reduced to ferrous tripyridyl triazine (Fe^2+^- TPTZ) by a reducing agent. In the 3% PPE extract, the FRAP value was determined to be 0.338 ± 0.010 (OD) at 1000 *μ*g/mL and was found to be lower in the control samples [0.133 ± 0.047 (OD)].

When measuring the reducing power of ferrous-ferricyanide (Fe^3+^) stabilizing free radicals by donating hydrogen to the ferric-ferricyanide, the 3% PPE extract and control were 0.482 ± 0.061 (OD) and 0.119 ± 0.011 (OD), respectively. Additionally, the reducing power of Cu^2+^ was greater in 10% PPE (0.349 ± 0.012) than in the control (0.216 ± 0.002), a pattern similar to that observed for phenolic contents and radical scavenging abilities.

The chelating activities of 0.1% PPE and control at 1000 *μ*g/mL were 89.81 ± 0.56% and 75.36 ± 1.70%, respectively, confirming that the reducing powers and chelating effects are obtained through various Fenton reactions. Also, the FRAP value and reducing power were similar to the DPPH radical scavenging activities and ABTS cation radical scavenging activity patterns, respectively, whereas the reducing power of Cu^2+^ was similar to the superoxide anion radical scavenging activity. However, chelating showed higher activity at lower concentrations of plum.

### 3.3. Antimelanogenic Properties

#### 3.3.1. Prunus Fermentation Extract Is Not Cytotoxic to B16F0 Cells

**Figure 3:**
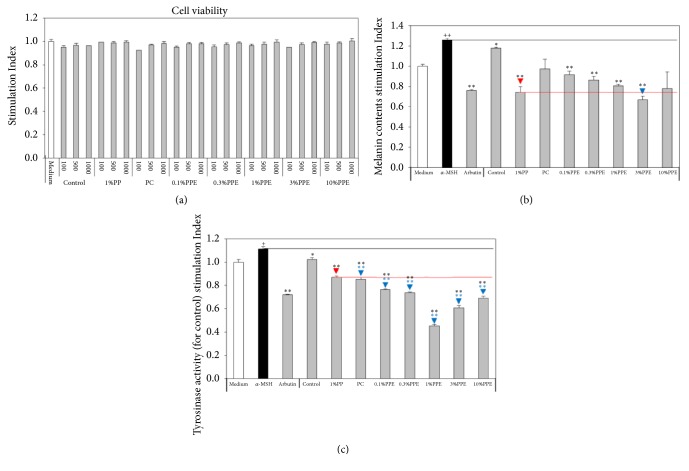
(a) Cytotoxicity of extract on mouse B16 melanoma cells. B16F0 cell line was treated with extracts for 24 hours and cell cytotoxicity was determined by CCK-8 assay. Culture supernatants were removed, and cell counting Kit-8 (CCK-8) was added. (b) Inhibitory effects of extract on the activity of tyrosinase. The lysates of B16F0 melanoma cells containing tyrosinase were incubated with DOPA for 1 h. Tyrosinase activity was measured as described in the Material and Methods. (c) Inhibitory effects of extract on the melanin synthesis in B16F0 melanoma cells. The cells were cultured in the presence of the extracts at concentration of 1000 *μ*g/mL for 48 h. The determination of melanin content was measured as described in the Materials and Methods. The results were expressed as the average of triplicate experiments. Data is expressed as a ratio of the control as mean±S.D of 3 separate experiments. ^+^p<0.05, ^++^p<0.01 or ^+++^p<0.001 Medium group vs. a-MSH group. *∗*p<0.05, *∗∗*p<0.01 or *∗∗*p<0.001 a-MSH group vs. Sample group. ^#^p<0.05, ^##^p<0.01 or ^###^p<0.001 1%PP group vs. PC, and PPE group.

We investigated whether different concentrations of the extract induce apoptosis in B16F0 cells. As shown in Figure 3(a), none of the extracts induce B16F0 cell cytotoxicity at any of the examined concentrations.

#### 3.3.2. Effects of Prunus Fermentation Extract on B16F0 Cells Melanin Synthesis Inhibitory Activity

Next, we evaluated the effects of the extracts on melanin synthesis in B16F0 cells, with an aim to evaluate potent antiwhitening properties. This was compared to the Prunus extract, which is known to exert an antiwhitening effect. Arbutin, a well-known inhibitor of melanin synthesis in B16F0 cells, was used as the positive control. As presented in Figure 3(b), the extracts significantly decrease melanin production in a dose-dependent manner corresponding to the content of Prunus in the medium. However, decreased melanin inhibition was observed in the medium containing 10% Prunus, thereby confirming that medium containing 3% Prunus is the optimal culture condition. The inhibitory action of medium containing 3% Prunus on melanogenesis was at a level equivalent to that of the standard arbutin.

#### 3.3.3. Effects of Prunus Fermentation Extract on the Tyrosinase Activity of B16F0 Cells

Tyrosinase is a well-known major regulator enzyme involved in melanin synthesis. Numerous inhibitors of melanin synthesis reduce melanogenesis by directly inhibiting the tyrosinase activity. The effect of the obtained extract on tyrosinase activity was assessed to tentatively evaluate their antimelanogenic properties since we identified that obtained extracts inhibited melanin synthesis. Likewise, arbutin, a well-known tyrosinase inhibitor, was used as a positive control. We further compared this with the Prunus extract, which is known to have an antiwhitening effect. We observed that exposure to the fermented extracts resulted in increased tyrosinase inhibition activities in the B16 melanoma cells (31%, 33%, 59%, 45%, and 37%) with increasing content of Prunus in the medium, as compared to cells treated with only Prunus (Figure 3(c)). Likewise, we confirmed that the optimal culture conditions were exhibited in the medium containing 3% Prunus.

## 4. Conclusions

In conclusion, we summarize the effects of extracts of the cultured* Poria cocos* mycelium fermented with freeze-dried plum powder (PPE) on *α*-melanocyte stimulating hormone (*α*-MSH)-stimulated melanogenesis in murine B16F0 melanoma cells (B16 cells), as compared with Prunus extract. Prunus fermentation extract showed significant inhibition of melanogenesis and tyrosinase, with no effect on cell proliferation. Furthermore, we confirmed that 3% PPE is the best culture condition for fermentation of* Poria cocos*. We also confirmed the antioxidant activity of 3% PPE. Similar patterns were seen in most experiments. Taken together, our results suggest that the extract is a functional antioxidant with potential for commercial application. These results provide evidence that 3% PPE affects skin whiting in murine B16F0 melanoma cells and can be provided as a potential mediator to induce skin whiting, as well as help in promoting the efficacy of antioxidants.

## Figures and Tables

**Table 1:** The chemical composition of cultured *Poria cocos* mycelium fermented culture extracts added freeze drying plum powder (PPE).

Type of Sample	Polyphenols (GAE)^a^	Tannins (CE)^b^	Flavonoids (QE)^c^
Control	2.120 ± 0.002	12.667 ± 0.002	1.286 ± 0.001
1% PP	2.887 ± 0.002	13.222 ± 0.001	2.476 ± 0.001
PC	2.212 ± 0.004	17.667 ± 0.001	2.714 ± 0.002
0.1% PPE	2.964 ± 0.001	14.889 ± 0.001	2.952 ± 0.001
0.3% PPE	3.323 ± 0.005	15.444 ± 0.001	2.714 ± 0.001
1% PPE	3.878 ± 0.003	18.222 ± 0.001	3.429 ± 0.001
3% PPE	4.701 ± 0.006	21.000 ± 0.003	5.333 ± 0.001
10% PPE	4.546 ± 0.006	18.779 ± 0.002	6.048 ± 0.001

Standard deviations (SD) did not exceed 5%, nd: not detected.

^a^Microgram Gallic acid equivalents per milligram. ^b^Microgram Catechin equivalents per milligram. ^c^Microgram Quercetin equivalents per milligram.

## Data Availability

The data used to support the findings of this study are available from the corresponding author upon request.

## References

[1] Chou S.-T., Chang W.-L., Chang C.-T., Hsu S.-L., Lin Y.-C., Shih Y. (2013). Cinnamomum cassia essential oil inhibits *α*-MSH-induced melanin production and oxidative stress in murine B16 melanoma cells. *International Journal of Molecular Sciences*.

[2] Seo S.-Y., Sharma V. K., Sharma N. (2003). Mushroom tyrosinase: recent prospects. *Journal of Agricultural and Food Chemistry*.

[3] Shirasugi I., Kamada M., Matsui T., Sakakibara Y., Liu M.-C., Suiko M. (2010). Sulforaphane inhibited melanin synthesis by regulating tyrosinase gene expression in B16 mouse melanoma cells. *Bioscience, Biotechnology, and Biochemistry*.

[4] Yasui H., Sakurai H. (2003). Age-dependent generation of reactive oxygen species in the skin of live hairless rats exposed to UVA light. *Experimental Dermatology*.

[5] Martínez-Esparza M., Jiménez-Cervantes C., Beermann F., Aparicio P., Lozano J. A., García-Borrón J. C. (1997). Transforming growth factor-*β*1 inhibits basal melanogenesis in B16/F10 mouse melanoma cells by increasing the rate of degradation of tyrosinase and tyrosinase-related protein-1. *The Journal of Biological Chemistry*.

[6] Jian D., Jiang D., Su J. (2011). Diethylstilbestrol enhances melanogenesis via cAMP-PKA-mediating up-regulation of tyrosinase and MITF in mouse B16 melanoma cells. *Steroids*.

[7] Hearing V. J., Jiménez M. (1987). Mammalian tyrosinase—the critical regulatory control point in melanocyte pigmentation. *International Journal of Biochemistry*.

[8] Kobayashi T., Imokawa G., Bennett D. C., Hearing V. J. (1999). Tyrosinase stabilization by Tyrp1 (the brown locus protein). *The Journal of Biological Chemistry*.

[9] Park H. Y., Kosmadaki M., Yaar M., Gilchrest B. A. (2009). Cellular mechanisms regulating human melanogenesis. *Cellular and Molecular Life Sciences*.

[10] Lin C.-C., Yang C.-H., Chang N.-F. (2011). Study on the stability of deoxyarbutin in an anhydrous emulsion system. *International Journal of Molecular Sciences*.

[11] Kang A. S., Kang T. S., Shon H. R. (1999). Studies on improvement of artificial cultivation and antioxidative activity of poria cocos. *The Korean Journal of Mycology*.

[12] Kim D. G., Son D. H., Kim S. M., Cho Y. S., Choi U. K. (2002). The antioxidant ability and nitrite scavenging ability of Poria cocos. *Journal of the Korean Society of Food Science and Nutrition*.

[13] Broadhurst R. B., Jones W. T. (1978). Analysis of condensed tannins using acidified vanillin. *Journal of the Science of Food and Agriculture*.

[14] Herald T. J., Gadgil P., Tilley M. (2012). High-throughput micro plate assays for screening flavonoid content and DPPH-scavenging activity in sorghum bran and flour. *Journal of the Science of Food and Agriculture*.

[15] Zhang Q., Zhang J., Shen J., Silva A., Dennis D. A., Barrow C. J. (2006). A simple 96-well microplate method for estimation of total polyphenol content in seaweeds. *Journal of Applied Phycology*.

[16] Kang K.-Y., Hwang Y.-H., Lee S.-J., Kim J.-J., Nam S.-J., Yee S.-T. (2017). Verification of the antioxidant activity of a subterranean part of Suaeda japonica Makino. *Industrial Crops and Products*.

[17] Blois M. S. (1958). Antioxidant determinations by the use of a stable free radical. *Nature*.

[18] Roberta R., Nicoletta P., Anna P., Ananth P., Min Y., Catherine R. E. (1999). Antioxidant activity applying an improved ABTS radical cation decolorization assay. *Free Radical Biology & Medicine*.

[19] Zhishen J., Mengcheng T., Jianming W. (1999). The determination of flavonoid contents in mulberry and their scavenging effects on superoxide radicals. *Food Chemistry*.

[20] Dinis T. C. P., Madeira V. M. C., Almeida L. M. (1994). Action of phenolic derivatives (acetaminophen, salicylate, and 5-aminosalicylate) as inhibitors of membrane lipid peroxidation and as peroxyl radical scavengers. *Archives of Biochemistry and Biophysics*.

[21] Gülçin I. (2007). Comparison of in vitro antioxidant and antiradical activities of L-tyrosine and L-Dopa. *Amino Acids*.

[22] Apak R., Güçlü K., Özyürek M., Esin Karademir S., Erçağ E. (2006). The cupric ion reducing antioxidant capacity and polyphenolic content of some herbal teas. *International Journal of Food Sciences and Nutrition*.

[23] Güçin I. (2008). In vitro prooxidant effect of caffeine. *Journal of Enzyme Inhibition and Medicinal Chemistry*.

[24] Benzie I. F. F., Strain J. J. (1996). The ferric reducing ability of plasma (FRAP) as a measure of "antioxidant power": the FRAP assay. *Analytical Biochemistry*.

[25] Göçer H., Gülçin I. (2011). Caffeic acid phenethyl ester (CAPE): Correlation of structure and antioxidant properties. *International Journal of Food Sciences and Nutrition*.

[26] Gülçin I., Elmastaş M., Aboul-Enein H. Y. (2007). Determination of antioxidant and radical scavenging activity of basil (*Ocimum basilicum* L. Family Lamiaceae) assayed by different methodologies. *Phytotherapy Research*.

[27] Gülçin I. (2010). Antioxidant properties of resveratrol: a structure-activity insight. *Innovative Food Science and Emerging Technologies*.

[28] Elmastaş M., Turkekul I., Öztürk L., Gülçin I., Isildak O., Aboul-Enein H. Y. (2006). Antioxidant activity of two wild edible mushrooms (Morchella vulgaris and Morchella esculanta) from North Turkey. *Combinatorial Chemistry & High Throughput Screening*.

[29] Bursal E., Köksal E., Gülçin I., Bilsel G., Gören A. C. (2013). Antioxidant activity and polyphenol content of cherry stem (Cerasus avium L.) determined by LC-MS/MS. *Food Research International*.

